# Sexual and Reproductive Health Challenges in Temporary Marriage: A Systematic Review

**DOI:** 10.34172/jrhs.2021.42

**Published:** 2021-01-18

**Authors:** Farzaneh Valizadeh, Abolfazl Mohammadbeigi, Reza Chaman, Fatemeh Kashefi, Ali Mohammad Nazari, Zahra Motaghi

**Affiliations:** ^1^Student Research Committee, Shahroud University of Medical Sciences, Shahroud, Iran; ^2^Research Center for Environmental Pollutants, Department of Epidemiology, Faculty of Health, Qom University of Medical Sciences, Qom, Iran; ^3^Department of Epidemiology, School of Health, Shiraz University of Medical Sciences, Shiraz, Iran; ^4^Department of Family Health, Deputy of Health, Mazandaran University of Medical Sciences, Mazandaran, Iran; ^5^Department of Reproductive Health, School of Nursing and Midwifery, Shahroud University of Medical Sciences, Shahroud, Iran; ^6^Reproductive Health Department, School of Nursing and Midwifery, Shahroud University of Medical Sciences, Shahroud, Iran

**Keywords:** Induced Abortion, Sexually Transmitted Diseases, Unplanned Pregnancy, Temporary Marriage, Violence

## Abstract

**Background:** Temporary marriage is a legal form of marriage in Shia Islam allowing a man and a woman to become married in a fixed period of time. This review was conducted to identify the potential effects of temporary marriage on the sexual and reproductive health of women.

**Study design:** A systematic review

**Methods:** Electronic databases, including Web of Knowledge, Embase, PubMed, Scopus, ScienceDirect, PsycINFO, ProQuest, IranMedex, Scientific Information Database (SID), and Magiran, were searched up to October 2020 to identify the studies carried out on sexual and reproductive health challenges in temporary marriage. All the selected articles were assessed for eligibility according to their titles and abstracts.

**Results:** During the search on articles published within 1995 to October 2020, 1,802 relevant records were identified, and after evaluation 30 full-text papers were included in the present systematic review. Out of the 34,085 study participants in the selected studies, 3,547 subjects had temporary marriage who were studied under six different categories, namely (1) sexually transmitted infections (STIs)/human immunodeficiency viruses, (2) early child marriage, (3) unplanned pregnancy and induced abortion, (4) violence, (5) psychosocial disorders, and (6) other issues. Individuals with temporary marriage are vulnerable and need to have easy access to health education and sexual and reproductive health services in a safe and unprejudiced environment. Ignoring the aforementioned facts will cause serious public health problems, especially for women from a lower socioeconomic background.

**Conclusions:** In the current situation with under-reporting of temporary marriage-related events due to social stigma and absence of quality services in sexual and reproductive health, women with temporary marriage are under the additional risk of STIs, unwanted pregnancy, abortion, and violence.

## Introduction

Temporary marriage or Mut’ah or Sigheh is a temporal contract between a man and an unmarried woman in which the couple agrees to be married for a speciﬁed length of time (from 1 hour to 99 years) for a ﬁxed sum of money or gift that is given to the woman. Although temporary marriage is a legal and legitimate form of marriage and permitted in Shia Islam, it is looked down upon by self-respecting families and is not acceptable in the community. Temporary marriage is legitimate in Iranian civil law (Principles: 1075, 1095-98) ^1-4^. Due to no necessity to register a temporary marriage and secrecy and social stigma of being involved in such type of marriage, no valid statistics are available on temporary marriage. However, unofficial sources report that the number of temporary marriages has been rising^5^.

Temporary marriage and other sorts of nonconventional marriages are observed within the extended Middle East and North Africa (EMENA) region, including Iran. Consistent with the results of previous studies, the EMENA region may be a region with the youngest population, where globalization, migration, information technology, and political changes are contributing to the reshaping of sexual behaviors and marriage ^6,7^. Countries with legal temporary marriage (e.g., Iran, Lebanon, and Iraq) are facing social and health concerns related to temporary marriage that may affect public sexual and reproductive health. Issues affecting temporary marriage health-related topics include under-reporting temporary marriage due to social stigma and limited reproductive and sexual health services (particularly sex protection and family planning services) ^8,9^.

In war-torn countries, children are even more at risk of under-aged forced and temporary marriage due to economic and social problems ^10,11^. This group of girls has contracted sexually transmitted infections (STIs) and about 14% of them were already human immunodeficiency viruses (HIV) positive ^12^. Some studies showed that the phenomenon of temporary marriage plays a role in the promotion of early child marriages in Iran ^13^.

These nonpermanent and often secret marriages are often a source of severe oppression and no sexual freedom or socioeconomic independence ^14^. However, according to several experts, although temporary marriage is a legitimate response to young individuals’ needs due to economic and social barriers to conventional marriage, it may threaten women’s health by increasing the risk of STIs/HIV, early child marriage, unwanted pregnancies, illegal abortions, coercion, and loss of family and social supports ^7,9,14^.

 In addition, due to its secrecy, temporary marriage does not provide women with legal protection against different types of abuse that most married women seek. This causes wives with temporary marriage to experience abuse, anxiety, limited social interactions, fewer social opportunities (i.e., occupational and educational opportunities), and therefore lower quality of life. Most published studies have been qualitative or based on legal jurisprudential aspects of temporary marriage. Health-oriented studies are paid less attention as they are scattered and discrete. Therefore, it is necessary to systematically review the health-oriented studies to provide a better and more realistic view of the effects of temporary marriage on women’s health.

## Error! Bookmark not defined. Methods

###  Protocol and registration

 The present systematic review was conducted in accordance with the Preferred Reporting Items for Systematic Reviews (PRISMA) statement. The protocol of systematic reviews is usually registered in the international prospective register of systematic reviews. This study was submitted to PROSPERO (receipt code: 226070).

###  Search strategy and study selection criteria 

 A comprehensive search was performed to identify any cross-sectional, cohort, or case-control studies investigating sexual and reproductive health challenges in women with temporary marriage. Accordingly, several sources, including Web of Science, Scopus, PubMed, PsycINFO, ProQuest, ScienceDirect, Magiran, Scientific Information Database (SID), IranMedex, and MedLib, were searched. Five sets of related Medical Subject Headings (MeSH) and nonMeSH terms in titles, abstracts, or keywords were used, including (“Temporary Marriage” OR “Sigheh” OR “Mut’ah”, OR “Short-form Marriage” OR “Short-term Marriage” OR “Nikah Mutʼah”) AND (“STD” OR “Sexually Transmitted Disease” OR “STI” OR “Sexually Transmitted Infection” OR “STI/ HIV” OR ”Venereal Diseases”) OR (“Reproductive Health”) OR (“Abortion” OR “Pregnancy Loss” OR “Miscarriage”) OR (“Violence” OR “Domestic Violence” OR “Spousal Abuse”, OR “Intimate Partner Violence”, OR “Sexual Violence” OR “Sexual Abuse” OR “Sex Offense” OR “Coercion”) OR (“Unplanned pregnancy” OR “Unintended Pregnancy” OR “Unwanted Pregnancy” OR “Mistimed Pregnancy”), and their equivalent in MeSH. Furthermore, a combination of these keywords was used for article extraction and searched for articles published up to October 2020. The search strategy was performed using Boolean operators (AND, OR). Two authors independently reviewed the articles and discrepancies were resolved by discussing with the third author. The reference lists of related articles were also manually reviewed for other possibly relevant studies that were not identified through the electronic search strategy.

###  Inclusion and exclusion criteria

 Observational, prospective or retrospective cohort, case-control, and cross-sectional studies investigating the temporary marriage were included in the present review. Duplicate publications or studies using the same sample were excluded. Studies not reporting sample size, review studies, qualitative studies, editorials, letters to the editors, commentaries, expert opinions, case series, case studies, brief reports, and book chapters were not included in the present systematic review and meta-analysis.

###  Data extraction and quality assessment

 Two independent authors (F.V and A.MB) extracted several characteristics from the included studies, such as author’s name, year of publication, study state/province, study sample size, and number of temporary marriages of individuals. Any disagreement between the two reviewers was resolved through discussion with the senior author (Z.M). The Newcastle-Ottawa scale (NOS) is one of the most known scales for the assessment of the quality and risk of bias in observational studies. The NOS checklist is assigned up to a maximum of nine points for the least risk of bias in three domains, namely selection of study groups (4 points), comparability of groups (2 points), and ascertainment of exposure and outcomes (3 points) for these studies, respectively. Based on the NOS, the quality of the articles was rated within a range of 0-9. Total scores were categorized into three following groups:

- (0-3): Very high risk of bias
- (4-6): High risk of bias
- (7-9): Low risk of bias ^15,16^

According to some sources for data retention, studies with lower than 5 points ^17^ or some studies with a score lower than the mean score of studies ^18^ identified as representing a high risk of bias were considered low quality and excluded ^17,18^. In this study, based on the NOS, studies with lower than 5 points were regarded as low quality and excluded. For each article, some information, including authors, objectives, places, sample sizes, study types, statistical analyses, and main results, were extracted. Each study was independently assessed by three authors (F.V, A.MB, and F.K) and then the data were independently extracted. Other authors (Z.M, R.C, and A.M.N.) were considered an arbiter to resolve any disagreements.

## Results

###  Included studies

**Figure 1:**
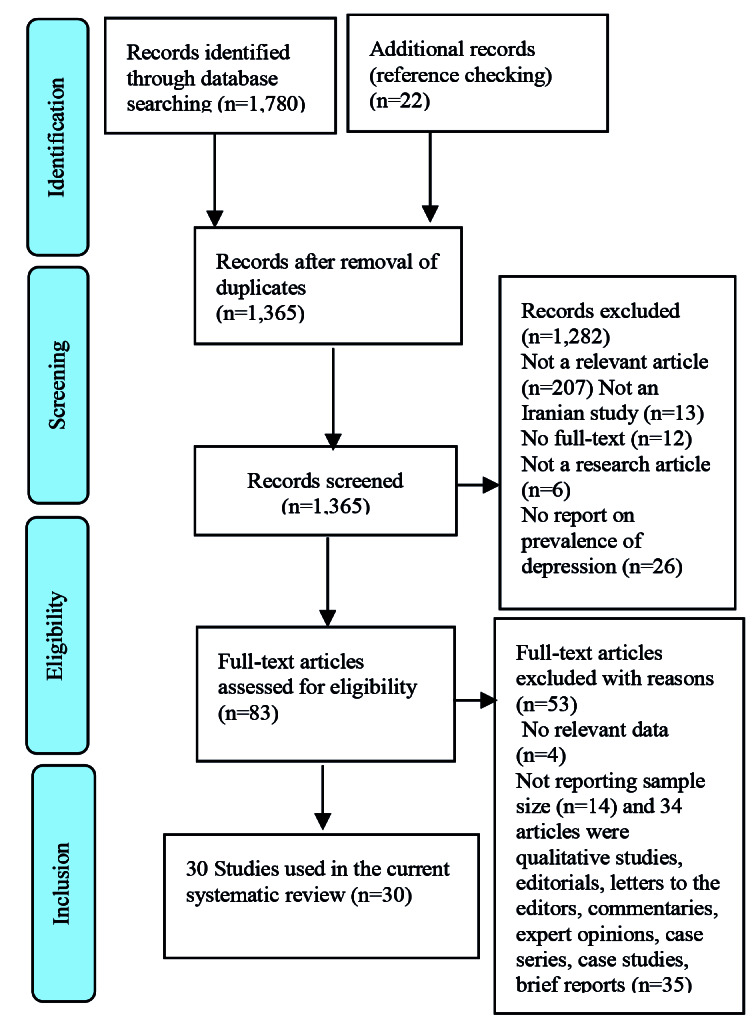
PRISMA flowchart of studies included and excluded at each stage of screening

As illustrated in Figure 1, according to the PRISMA flowchart, 1,802 studies which were reported by eight countries were extracted through searching. Out of these 1,802 studies, 1772 papers were excluded after reviewing the titles and abstracts, then full text of the remaining articles were assessed. Finally, 30 studies met the eligibility criteria and were included in the current systematic review. In the present study, no study was identified on the health consequences of temporary marriage, except for 28 Iranian articles and 2 articles on Syrian refugees in Jordan. There were 30 relevant articles that were published within 1998 to October 2020 with 34,085 study participants, out of whom 3,547 individuals had temporary marriage. According to these studies, the rate of temporary marriage was various in different areas and groups. Table 1 tabulates the main characteristics of the included studies. The selected (n=30) articles were published within 1998 to October 2020 in six different categories, namely 1) STIs/HIV, 2) early child marriage, 3) abortion and unintended pregnancy, 4) violence and coercion, 5) psychosocial disorders, and 6) others issues.

###  STIs/HIV 

Through searching, 18 articles were identified on STIs/HIV among individuals with temporary marriage. Based on the sampling methods, these articles were divided into two study groups. In the first group (n=5; with a sample size of 8,293 individuals), the studied population consisted of individuals of reproductive age. The results of studies carried out by Khani et al. and Rostami et al. on women referring to health care centers in northern cities of Iran, Hassani et al. on volunteers for marriage in Bandar Abbas, Iran, Shokoohi et al. on individuals within the age range of 15-29 years at a national level, and Kazem Mohammad et al. on male adolescent aged 15-18 years in Tehran, Iran, showed that temporary marriage experience and unprotected sex were within the ranges of 1.5-14% and 50-80%, respectively ^19-23^. In the second group (n=13), high-risk behavior groups, including female sex workers and male intravenous-drug users, with a sample size of 7,691, were studied at a national level. The results indicated that a significant percentage of individuals in these high-risk groups had at least a temporary marriage and the rates of unprotected sex and STIs were very high ^24-36^. The results of logistic regression in a study performed by Fallahi et al. demonstrated that the risk of acquiring STIs increases 4.3 times in homeless women with temporary marriage, compared to that reported for homeless women without temporary marriage (OR=4.33; OR=3; 95% CI: 1.39-13.49; *P*=0.010) ^28^. In a study conducted by Shaditalab et al., it was estimated that 30% of female addicts in Tehran had multiple temporary marriages ^37^. In addition, a study carried out on Syrian refugee women showed that 11.5% of women with temporary marriage reported having STIs and two-third of them declared that their spouses refused to use a condom during their sexual intercourse ^38^.

‬‬‬‬‬‬‬‬‬‬‬‬‬‬‬‬‬‬‬‬‬‬‬‬‬‬‬‬‬‬‬‬‬‬‬ ***Early child marriage***

The International Planned Parenthood Federation defined early child marriage as any marriage under the age of 18 years ^12^. Iran Demos and Health Statistics states that in some parts of Iran temporary marriage among under-age girls (not reaching the legal age for marriage) is common. In other words, temporary marriage leads to child abuse by spouses ^13^. In searching with the related keywords, a study conducted on temporary marriage in young females among Syrian refugees was identified. According to the results, young-female and older Syrian refugees were sexually exploited through the temporary marriage system. In 2013, approximately 25% of the registered temporary marriages of Syrians in Jordan involved Syrian adolescents within the age range of 15-17 years and the majority of these children were girls ^39^.

###  Unplanned pregnancy and abortion

Unwanted pregnancies and their complications are the most important factors with a negative impact on women’s health. Studies carried out by Cheraghi et al., Motaghi et al., and Francome on the complications of women’s health with temporary marriage indicated that Nikah mutʼah makes the institutionalization of sex without commitment possible. In addition, mutʼah is considered one of the most common predictive factors of unwanted pregnancies and unsafe abortions that is usually due to a men’s lack of participation in using family planning methods ^40-42^. Abortion is a multidimensional phenomenon with several social, legal, and religious implications ^43^. The results of a study conducted by Pourreza and Batebi showed that 6% of women referring to medical centers for abortion had temporary marriage and were at risk for physical and mental complications due to a lack of sociocultural acceptance ^44^. Moreover, the results of a study carried out by Jamshidi and Saroukhani in obstetrics and gynecology clinics of Alborz province, Iran, indicated that 33% of women with temporary marriage had a history of unwanted pregnancy, 88% of whom had abortions and 22.7% of whom were left by temporary husbands ^45^. Similarly, a study performed by Nokhodian et al. in Isfahan, Iran, showed that 36% of female prisoners with temporary marriage had a history of abortion ^27^. In this regard, the results of a study performed by Jahanfar et al. on women referring for antenatal care demonstrated that 0.7% of them were women with temporary marriage ^46^. The results showed that most women with temporary marriage were reluctant to continue the pregnancy because their husbands abandoned them and they reported different supportive problems.

**Table 1:** Main characteristics of the studies included in the present systematic review

**No.**	**Author, y ear**	**Target group, country**	**Age (yr )**	**Sampling method**	**Type of study**	**Results**	**N OS**
**STIs/HIV**
1	Zargooshi et al, 2002 ^31^	100 male gonorrhea patients, Iran	19-35	Convenience sampling	Cohort	24 men became infected by temporary wives (100% - temporary marriage-individual) and78% of males had unprotected sex (n=19)	7
2	Ghanbarzadeh el al, 2005 ^33^	200 women prisoners, Iran	16-73	Census method	Cross-Sectional	22 women reported that they had temporary marriage and prevalence of STI among them was 11%.	5
3	Kazem Mohamad et al, 2007 ^22^	1,385 adolescent males, Iran	15-18	Cluster sampling	Cross-Sectional	198 (14.2%) adolescent boys had sexual contact with women with temporary marriage 164 (37%) of their partners had protected sex.	9
4	Tehrani et al, 2008 ^26^	At-risk-1005 Young, Iran	15-40	Segmentation method	Cross-Sectional	379 (37%) youths had temporary marriage; prevalence of STIs and unprotected sex were 34% and 35.1%, respectively.	8
5	Kassaian et al, 2011 ^29^	100 female sex workers, Iran	18-42	Convenience sampling	Cross-sectional	34 female sex workers reported having temporary marriage; 16% of their partners had protected sex and 9 % of them had HCVAb+	6
6	Nokhodian et al, 2012 ^27^	163 women prisoners, Iran	15-45	Census method	Cross-sectional	29 of female prisoners reported temporary marriage and having HCVAb (7%).	7
7	Alipour et al, 2013 ^27^	452 male and female intravenous-drug abusers, Iran	20-41	Convenience sampling	Cross-sectional	88 (%19) subjects reported having temporary marriage; %27 of their partners had protected sex.	8
8	Hassani Azad et al, 2016 ^23^	600 volunteers for marriage, Iran	19-34	Available sampling	Cross-sectional	12 men and women reported having temporary marriage 55% of whom had unprotected sex and did not have HIV positive test.	7
9	Shokoohi et al, 2016 ^24^	1,005 female sex workers, Iran	25–34	Facility-based sampling	Cross-sectional	200 had temporary marriage; 17% of them were HIV-positive and 64% of them had received free condoms.	8
10	Shokoohi et l, 2016 ^21^	5,395 youths, Iran	15-29	Multistage cluster sampling	Cross-sectional	562 youths had temporary marriage; 21.8% of their partners had protected sex.	9
11	Rostami et al, 2017 ^20^	400 women referring to health centers, Iran	20-36	Available sampling	Cross-sectional	12 of women referring to health centers had temporary marriage; 70% and 65% of them had unprotected sex and STIs, respectively.	7
12	ShahEsmaili et al, 2017 ^32^	1,337 female sex workers, Iran	23-44	Facility-based sampling	Cross-sectional	134 (10%) FSW reported having temporary marriage; 50% and 32.9% of women with temporary marriage had STI and protected sex, respectively; the most prevalent STIs was human papillomavirus, and prevalence of HIV was 2.0%.	8
13	Rezaianzadeh et al, 2017 ^25^	1,052 individuals infected with HIV, Iran	25-49	Census method sampling	Historical cohort	16 (1.5%) HIV^+^ subjects reported having temporary marriage.	6
14	Khoei et al, 2017 ^35^	300 Iranian male drug users, Iran	31-38	Segmentation method	Cross-sectional	5 (1.5%) drug users had a history of temporary marriage 80% of whom had high-risk sexual behaviors.	8
15	Khani et al, 2018 ^19^	514 female sex workers, Iran	23-38	Multistage sampling	Cross-sectional	13 (2.6%) women had the experience of temporary marriage 85% of whom did not use a condom; 56% of them had STIs.	7
16	Behzadi et al, 2018 ^30^	71 HIV-infected women, Iran	27-36	Convenience sampling	Cross-sectional	5 (7%) women reported having temporary marriage; 21% and %77 of women with temporary marriage had protected sex and other STIs.	8
17	Asadi et al, 2018 ^34^	184 female sex workers, Iran	23-40	Convenience sampling	Cross-sectional	27 FSW reported having temporary marriage 56% of whom had unprotected sex.	7
18	Fallahi et al, 2019 ^28^	241 homeless women in DIC, Iran	20-48	Quota sampling	Cross-sectional	57 (23.5%) homeless women reported having temporary marriage; 23% and 22% of women with temporary marriage had STIs and protected sex, respectively; the results of logistic regression showed that the risk of acquiring STIs increases 4.3 times in homeless women with temporary marriage, compared to that of homeless women without temporary marriage (OR=4.33; OR=3; 95% CI: 1.39-13.49; P=0.01).	8
19	Dua’ Al-Maharma et al, 2019 ^38^	523 Syrian refugee women, Jordan	16-44	Proportional quota sampling	Cross-sectional	11.5% of Syrian refugee women with temporary marriage (n=60) had STIs and 67% of them had unprotected sex.	7
**Early child marriage**
20	Spencer RA, et al 2015 ^39^	2,936 registered Syrian temporary marriages, Jordan	15-17	Census method	Cross-sectional	Syrian young girls and women are sexually exploited through a temporary marriage; 25% of them (n=735) involved Syrian girls within the age range of 15-17 years.	6
**Unintended pregnancy**
21	Jamshidi et al, 2016 ^45^	53 women with temporary marriage, Iran	21-40	Convenience sampling	Cross-sectional	33% of women with temporary had unintended pregnancy; 88% of them aborted their pregnancy.	7
22	PourReza et al, 2011 ^44^	278 women with abortion, Iran	15-49	Convenience sampling	Cross-sectional	6% (17) of women who had abortion experiences were women with temporary marriage.	8
23	Jahanfar et al, 1998 ^46^	3,028 women seeking prenatal care, Iran	18-41	Proportion stratification	Cross-sectional	Only 0.7 % of subjects reported having temporary marriage.	7
Repeated	Nokhodian et al, 2012 ^27^	163 female prisoners, Iran	15-45	Census method	Cross-sectional	29 (18%) female prisoners reported temporary marriage; 36% of women with temporary marriage had an abortion.	8
**Violence**
24	Eslamloo et al, 2006 ^48^	261 women with spousal abuse referring to Urmia Forensic Medicine, Iran	21-46	Census method	Cross-sectional	60 (23 (%women complaining to forensic medicine were women with temporary marriage; Adjusted OR: 4.6 (CI 95%:1.25-17.7; P: 0.01) of violence in temporary marriage in comparison to that in permanent marriage	7
25	Khoei et al, 2015 ^50^	120 female drug users, Iran	26-45	Multistage sampling	Cross-sectional	0.8% of female drug users reported having temporary marriage and experienced high rates of domestic violence.	8
26	Rahmani et al, 2020 ^49^	1,337 femal sex workers, Iran	20-45	Facility-based sampling	Cross-sectional	16.5% (220) of female sex workers reported having temporary marriage and 18.2% (40) of them had experiences HAS.	7
Repeated	Khani et al, 2018 ^19^	514 women referring to health care centers, Iran	23-38	Multistage sampling	Cross-sectional	13 (2.6%) women had the experience of temporary marriage; (30%) of them experienced sexual coercion.	7
Repeated	Dua’ Al-Maharma, et al, 2019.^38^	523 Syrian refugee women, Jordan	15-44	Proportional quota sampling	Cross-sectional	6.5% of women with temporary marriage experienced domestic violence and 9.1% of them suffered from sexual violence.	7
**Psychosocial disorders**
27	Zarei et al, 2017^51^	150 divorced women, Iran	27-36	Convenience sampling	Cross-sectional	2.6% of divorced women reported having temporary marriage; the use of the Social Exclusion Questionnaire in Iranian divorced women indicates that women with temporary marriage had psychosocial effects.	8
28	Salarifar, et al, 2015 ^52^	60 householder women with temporary marriage and 60 householder women without temporary marriage, Iran	28-38	Random sampling	Case/Control	The mean score of General Heath Questionnaire (GHQ)-test and Rif-test (Rehabilitation Psychological) indicated that the householder women with temporary marriage were better than householder women without temporary marriage indicating that the psychological and emotional state of women with temporary marriage is better.	7
29	Ahmadi et al, 2011 ^53^	1,025 Iranian soldiers, Iran		Cluster sampling	Cross-sectional	2% of these soldiers reported having temporary marriage and tendency toward alcohol and multi-substance abuses.	6
Repeated	PourReza et al, 2011 ^43^	278 women with abortion, Iran	15-49	Convenience sampling	Cross-sectional	33.4% of women with temporary marriage had an abortion and experienced psychological side effects.	8
**Other issues**
30	Valizadeh et al, 2016 ^54^	20 couples who were married without going to marriage center and without being screened for thalassemia, Iran	16-30	Convenience sampling	Cross-sectional	25% of couples had temporary marriage and minor couple marriage.	7

**STIs: **sexually transmitted infections; **HIV:** human immunodeficiency viruses; **DIC**: drop-in center; **HAS**: heterosexual anal sex; **FSW: **female sex workers **; HCVAb :** hepatitis C virus antibody

###  Violence

Women with temporary marriage are at higher risk of violence and spousal abuse, compared to other women ^47^. In the present review study, four studies were identified in this regard. The results of a study carried out by Eslamlou and Boshehri on women with spousal abuse referring to the Urmia Forensic Medicine Center, Iran, indicated that about 20% of the female complainants were women with temporary marriage. Domestic violence in females with temporary marriage was compared to that reported for women with permanent marriages (Adjusted OR: 4.6; CI 95%: 1.25-17.7; P=0.01). The authors suggested temporary marriage as the strongest risk factor for violence and spousal abuse ^48^. Furthermore, a study conducted by Rahmani et al. demonstrated that 18.2% of women with temporary marriage had a lifetime experience of heterosexual anal sex ^49^. The results of a study conducted by Merghati-Khoie et al. showed that addicted women with temporary marriage faced violence and all kinds of physical and sexual abuse, and condom use was positively associated with harassment and psychological abuse of women ^50^. A study carried out by Khani and Moghadam in the north of Iran indicated that the main reason for these women’s referral to medical centers was sexual coercion and 85% of the participants in the aforementioned study also reported that their partners did not use a condom during intercourse. In fact, neglecting the health status and sanitary needs of women with temporary marriage is considered spousal abuse ^19^. The Syrian young-female and older refugees in Jordan are sexually abused through a temporary marriage. About 6.5% of these refugees experienced spousal abuse and 9.1% of them had sexual abuse of any type, including coercion, forced intercourse, and anal sex during menstruation ^38^.

###  Psychosocial disorders

Four studies were identified in this search with regard to psychosocial disorders. The results of a study carried out by Zarei et al. on divorced women with temporary marriage using the Social Exclusion Questionnaire in Iranian divorced women suggested that these women suffered from mental disorders, such as anxiety, depression, and psychosomatic disorders, due to a lack of social and economic support during and after the end of temporary marriage. Therefore, they have to look for a new relationship or an extension to their previous temporary marriage ^51^. A study conducted by Pourreza and Batebi showed that 33.4% of women with temporary marriage had an abortion and experienced psychological side effects ^44^. Another study performed by Salarifar demonstrated that the head of household with temporary marriage had a better mental, emotional, sexual, and physical status than the head of household without temporary marriage ^52^. Moreover, Ahmadi et al. observed that temporary marriage was one of the predictive factors for drug abuse among young individuals ^53^.

###  Other issues

In addition to the five above-mentioned topics, there are other issues. For example, the findings of a study carried out by Valizadeh et al. showed that out of 20 couples who got married without going to the marriage center and without being screened for thalassemia, 5 couples had temporary marriage and minor couple marriage ^54^.

## Discussion

In this systematic review, it was observed that STIs were very common in women with temporary marriage. The main causes of the increase in the prevalence of STIs among this population in different studies were the absence of trained health care and misjudgment by providers in reproductive and sexual health services, under-reporting, and poor notifications due to social stigma, causing them to be an important potential source of STIs, especially treatment-resistant STIs ^28,55^. Furthermore, unprotected sex and lack of men’s participation in the use of barrier methods in sexual relations neglecting the health of women with temporary marriage and spousal abuse were other causes of the increase in the prevalence of STIs among this population ^19,47^.

In sexual violence, victims are vulnerable to sexual and reproductive health consequences, such as unwanted pregnancies, unsafe abortions, and higher risk of STIs in unprotected sex^38^. Barriers to condom use seem to be socioculturally determined by gender stereotypes. In addition, stigma leads to a lack of control over the sources and culture of patriarchy in society, gender inequity, and lack of womenʼs negotiation power with men. These cause many problems, including less access to health care, economic dependency, and ultimately citizenship needs, among women with temporary marriage which are kinds of violence against women ^32,56,57^. Spousal abuse or intimate partner violence as a health and social problem is accompanied by multiple physical and sexual, reproductive, and psychological health complications, poor quality of life, social consequences, and even mortality ^47,34^.

On the other hand, one of the main reasons for abortion is temporary marriage. These marriages are not socially accepted and often remain secret from the couplesʼ families and community. This limits couples’ access to reproductive health services and may increase the risk of unintended pregnancy^40,58^. Temporary wives are forced to accept unsafe and illegal abortions due to a fear of stigmatization, prejudiced health care services, and absence of active services in sexual and reproductive health. Unsafe abortion is the third leading cause of maternal mortality and affects the physical, emotional, and social health of women and judicial ^43^. Temporary marriage in countries in which it is prevalent is one of the strong predictors of unwanted pregnancy and unsafe abortion ^40,59^ due to the confirmation and proof of the father-child lineage, despite reliable experiments, is still difficult due to its high cost and lack of access ^60^.

In addition, it is reported that some female adolescents who have not reached the legal age of marriage are forced to accept temporary marriage by their families or caregivers. The average rate of early marriage was reported as 16%, with 23% in Baluchestan as the highest rate in Iran ^13^. When brides are much younger than their spouses, the gap in age will make them in a weaker position in case they need to express their physical, emotional, and health care needs that in turn cause more serious reproductive health complications, such as unwanted pregnancy, abortion, preterm labor, and fetal mortality ^12,13^. In war-torn countries (e.g., Algeria, Sudan, Morocco, and Syria ), hundreds of women and girls have been sold under the guise of temporary marriage or girls are abducted or kidnapped by armed militia or rebels and forced into temporary marriage as a combination of child prostitution and pure slavery ^10,61^. Syrian families in asylum countries have a disturbing sense of insecurity, vulnerability, and real and perceived risk of sexual harassment. Early marriage under the guise of protecting girls against poverty and insecure conditions saves their families from financial constraints ^38,61^.

In several studies, it was indicated that women with temporary marriage mostly suffered from psychosocial issues causing two types of concerns including 1) intrapersonal communication disorders (e.g., anxiety, depression, and lack of self-esteem) and 2) interpersonal communication disorders (e.g., a lack of love and empathy) ^61,62^. Numerous studies reported STIs, sexual abuse, unwanted pregnancy, and abortion as several possible outcomes of temporary marriage ^63^. Finally, the existing stigma to temporary marriage has made this group extremely hard-to-access by the health care providers. It should be considered to address the sexual and reproductive health needs of women with temporary marriage. This paying attention to the sexual and reproductive health needs of this group of women not only protects their right to live longer and healthy lives but also is an important step toward maintaining the health of society. In fact, temporary marriage has made the vision of womenʼs rights and reproductive-sexual rights more complicated ^19^.

###  Limitations

Throughout the world, most of the studies carried out on temporary marriage have been based on legal and jurisprudential approaches or qualitative studies; however, this topic with a health-oriented approach has been paid less attention. Culturally, the Middle East is a conservative society in which sexuality, sexual health issues, and temporary marriage are social taboos and women with temporary marriage due to a fear of stigmatization and social misjudgment avoid from undergoing medical care and are unaware of available or sufficient services ^38^. Embarrassment would be one cause for concealing some relevant and important relationship information. For these reasons, studies conducted on women with temporary marriage with a health-oriented approach were under-reported and limited in number. Moreover, the use of nonrandom sampling restricted the generalizability of the findings and increased information bias and selection bias. Self-administered questionnaires provide a more private and less threatening means of reporting sensitive behaviors, thereby reducing information and reporting or recall bias or Sensitive study method can be used.

## Conclusions

Temporary marriage is proposed as a legitimate sexual relationship between a man and a woman. However, the secrecy, lack of knowledge about the properties of temporary marriage among young girls or women, and lack of legal and organized social support for the temporary wives make this group socially, mentally, and physically vulnerable. Ignoring these concerns can put temporary wives at a higher risk of health, family, and social problems ^50^. The absence of quality resources and services in reproductive and sexual health, under-reporting temporary marriage and its side effects, and lack of governmental and social support due to social stigma contribute to risky illegal and induced abortions, violence, early child marriage, and STIs among women with temporary marriage. Therefore, it is recommended to provide women with temporary marriage in legal age with legal and social supports, expand access to health education and sexual and reproductive services, and review womenʼs rights and reproductive-sexual health rights.

## Acknowledgements

 The present study was supported by Shahroud University of Medical Sciences as a PhD thesis (grant no.: 98119). Hereby, the authors would like to express their gratitude to the Research Deputy of Shahroud University of Medical Sciences.

## Conflict of interest

 The authors declare that there is no conflict of interest.

## Funding

 The present study was supported by Shahroud University of Medical Sciences, Iran (code: 98119).

## Highlights

- Temporary marriage is a legitimate sexual relationship; however, it can be a threat to individuals and social health.
- In this review, the potential effects of temporary marriage were sexually transmitted infections, unwanted pregnancies, abortions, violence, psychosocial disorders, and early marriage.
- Wives with temporary marriage have an urgent need for sexual and reproductive health services in a safe and unprejudiced environment.
